# Laterality as a Tool for Assessing Breed Differences in Emotional Reactivity in the Domestic Cat, *Felis silvestris catus*

**DOI:** 10.3390/ani9090647

**Published:** 2019-09-03

**Authors:** Deborah L. Wells, Louise J. McDowell

**Affiliations:** Animal Behaviour Centre, School of Psychology, Queen’s University Belfast, Belfast BT7 1NN, UK

**Keywords:** animal welfare, breed, cats, feline, laterality, motor bias, paw preferences

## Abstract

**Simple Summary:**

Cat breeds differ enormously in their emotional reactivity, a factor that can impact upon the success of the pet-owner relationship, with indirect consequences for animal welfare. Traditional methods of assessing emotional reactivity in cats have focused largely on questionnaire-based assessments of breed-specific behavioural profiles. We explored whether paw preferences, which have been linked to emotional reactivity in animals, are related to cat breed. The paw preferences of 4 commonly owned cat breeds were tested on a food-reaching challenge. Cats’ paw preferences differed between the breeds. Bengal cats were more likely to show a left-sided paw preference than other breeds, whilst Persians showed the weakest paw preferences, veering more heavily towards ambilaterality. Results confirmed our earlier work in showing a strong tendency for left paw use in male cats and right paw use in females. We propose that paw preference measurement could provide a useful method for assessing emotional reactivity in domestic cats, adding to our currently limited artillery of tools for determining breed-specific profiles. Such information would be of benefit to individuals considering the acquisition of a new cat, and, in the longer term may help to foster more successful cat-owner relationships, leading to indirect benefits to feline welfare.

**Abstract:**

Cat breeds differ enormously in their behavioural disposition, a factor that can impact on the pet-owner relationship, with indirect consequences for animal welfare. This study examined whether lateral bias, in the form of paw preference, can be used as a tool for assessing breed differences in emotional reactivity in the cat. The paw preferences of 4 commonly owned breeds were tested using a food-reaching challenge. Cats were more likely to be paw-preferent than ambilateral. Maine Coons, Ragdolls and Bengals were more likely to be paw-preferent than ambilateral, although only the Bengals showed a consistent preference for using one paw (left) over the other. The strength of the cats’ paw use was related to cat breed, with Persians being more weakly lateralised. Direction of paw use was unrelated to feline breed, but strongly sex-related, with male cats showing a left paw preference and females displaying a right-sided bias. We propose that paw preference measurement could provide a useful method for assessing emotional reactivity in domestic cats. Such information would be of benefit to individuals considering the acquisition of a new cat, and, in the longer term, may help to foster more successful cat-owner relationships, leading to indirect benefits to feline welfare.

## 1. Introduction

Lateralised motor behaviour has been studied as an observable measure of cerebral functional asymmetry for numerous years [1,2]. The most prominent manifestation of lateralised behaviour in humans is that of handedness (i.e., the predominant use of one hand), with roughly 90% of people using their right hand for most activities [3,4].

Studies now suggest that cerebral functional asymmetry is not unique to humans but may be a fundamental feature of all vertebrate, and even some invertebrate, brains (for reviews [5,6,7,8,9,10,11]). What is less clear is whether non-human species exhibit lateralisation in their limb use in a manner that approximates human handedness or whether the preferred use of a specific hand, paw or similar appendage is related to other aspects of brain asymmetry (see reviews [12,13,14]). Whilst there is a general consensus that individual animals may show consistent hand/paw preferences, the question of whether motor lateralisation exists at the level of the population remains controversial [15,16]. Population-level asymmetries have been found in a number of non-human species, including primates [17,18], humpback whales [19] and parrots [20], but studies on other species, for example, sheep [21,22,23], horses [24,25,26], dogs, (for review [27]), cats [28,29,30,31] and some insects [32,33], point more towards motor asymmetries at the level of the individual.

Limb preferences in animals may be related to a wide range of individual differences, including, for example, temperament [28,34], cognitive bias [35,36], sex [29,30,31,37,38]. Although subject to very little investigation, there are hints that motor laterality may also be related to breed. McGreevy and Thomson [39] found that Standardbred horses (animals bred for pacing) and Thoroughbreds (animals bred to race at the gallop) are more strongly left-footed than Quarter horses (animals bred to manoeuvre cattle), which, by contrast, are more likely to display ambilateral limb preferences. Although the influence of environmental factors cannot be ruled out, the authors argue that certain breeds of horse may perhaps have been unintentionally bred for particular lateralised behaviours as a result of the work they are required to carry out. More recently, the relationship between breed and motor asymmetry has been explored in the domestic dog, another species that has been selected for by humans to serve specific functions. McGreevy and colleagues [40] recorded the paw preferences of four dog breeds (two long-muzzle breeds—whippets, greyhounds—and two short-muzzle breeds—boxers, pugs) on the commonly employed Kong ball test. Findings showed no significant breed differences in the expression of motor bias on this task, although performance differences emerged between breeds; both of the short-muzzle breeds were quicker to reach the criterion of 100 paw responses than the long-muzzle breeds, which, by contrast, employed their paws less frequently, relying more heavily on their muzzle alone to retrieve the food.

The following study explores the influence of breed on motor bias in the domestic cat, a species that has been shown to display lateralised paw preferences at the level of the individual [28,29,30,31]. Unlike horses and dogs, cats have not been selected for by humans to serve any particular function. Yet, feline breed differences in behavioural phenotype (e.g., temperament) have been identified [41,42,43]. Whether breed differences in motor bias exist, however, is still unknown and in need of investigation [28], particularly from a welfare perspective. Research points to a strong link between limb use and emotional functioning in animals, with emotionally reactive individuals showing a significant leaning towards ambilaterality or left limb use (reflecting the dominant use of the contralateral right hemisphere), and more emotionally stable individuals favouring the use of their right limb (reflecting the dominant use of the left hemisphere). Studying the paw preferences of different breeds would provide useful information on genetic differences in emotional functioning in cats and would add to our existing knowledge base on breed-specific profiles for this species. Such information would be a useful tool for people considering the acquisition of a new cat. Pet-owner relationships frequently break down because of a discrepancy between owner expectations and the reality of pet ownership [44,45]. Prior knowledge on breed-related emotional functioning and behavioural disposition may help to reduce this disparity and foster more successful partnerships; indirectly, this may serve to promote feline welfare, e.g. via reduced relinquishments arising from dissatisfaction with the animal.

In the following study, the paw preferences of four commonly owned breeds (Ragdoll, Maine Coon, Persian, Bengal) were assessed using a previously employed measure of feline motor bias [28,29]. The breeds selected for study were deliberately chosen because of their different behavioural profiles, with two of the breeds (Ragdoll, Maine Coon) considered by veterinary practitioners to be emotionally non-reactive, and two of the breeds (Persian, Bengal) considered to be more emotionally labile [41]. It was anticipated that the research would elucidate whether these breed-related differences in emotional reactivity are reflected in the measurable outcome of motor bias and, more generally, would shed light on the relationship between breed and paw preferences in a species that has thus far been completely overlooked in this respect.

## 2. Materials and Methods

### 2.1. Subjects

Fifty-six purebred cats (15 Ragdoll, 15 Maine Coon, 14 Persian and 12 Bengal), aged between 1 and 9 years (2.82 ± 0.28 years) were tested. The sample was comprised of 24 (42.9%) males (66.7% neutered) and 32 (57.1%) females (59.4% neutered). Castration status did not differ significantly by either feline sex (*p* = 0.78, Fisher’s exact test) or breed (χ2 = 6.36, df = 3, *p* = 0.09). Animals were recruited following an email advertising a study on feline paw preferences, which was sent to staff and students in the School of Psychology, Queen’s University Belfast. Contact was also made with breeders at cat shows across the Province. All of the cats were family pets living in households whose owners had consented to them participating in the study. None of the cats had undergone any behavioural training nor had any disability or behavioural problem preventing them from completing the study.

### 2.2. Food-Reaching Test

**Figure 1:**
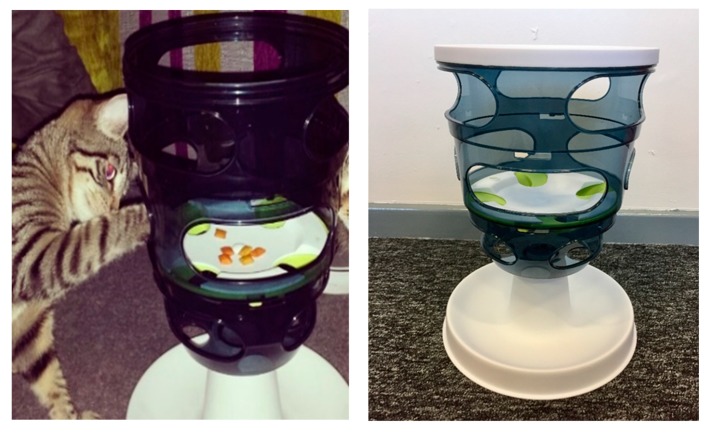
The Catit Senses Food Maze used to assess paw preferences.

Cats’ paw preferences for food reaching were assessed using the Catit Senses Food Maze (Catit, UK). This maze is a 35.6 cm-tall spherical feeding ‘tower’, comprising three levels into which food can be placed. The food is accessed via three holes on each level (see Figure 1). For the purpose of this study, the top level of the food maze was removed as it proved too high for the cats to put their paws into without standing on their rear legs and compromising balance. The Catit has been used successfully to record cats’ paw preferences [25,26] and demonstrates good test–retest reliability [46].

### 2.3. Procedure

All of the subjects were tested individually in their own home environment. The environment surrounding the food maze was made as symmetrical as possible to avoid influencing directional bias. Prior to the start of the first trial, the cat was shown, and allowed to sniff, the food treat (Dreamies, Mars Petcare, UK—small squares of cheese-flavoured cat treats). As the cat watched, 10 treats were placed on the top (i.e., second) level of the food maze. Since the food could be accessed through three holes, it was ensured that the cat was placed directly in front of one hole for each trial. The experimenter stood 1 m directly behind the cat throughout to avoid influencing the animal’s response. As and when required (i.e., once all the food treats had been accessed by the cat), another 10 treats were placed in the food maze. A single trial comprised the cat placing its paw inside the food maze and attempting to remove the treat. Only once the paw had been removed from the maze did the next trial begin. The paw the cat used to attempt food retrieval, regardless of whether or not a treat was obtained, was recorded. Each cat was tested until 50 responses were made. No human reward, be it tactile (comforting, stroking) or social (verbal praise or smiling), was provided during the task. The apparatus was cleaned thoroughly between testing with different subject animals.

### 2.4. Statistical Analysis

Binomial z-scores were calculated to determine whether the frequency of right- or left-side use exceeded that expected by chance alone. An alpha value of 0.05 was adopted. A z-score greater than +1.96 (two-tailed) reflected a significant left paw preference, whilst a z-score less than −1.96 indicated a significant right paw preference. Cats with z-scores between +1.96 and −1.96 were classified as ambilateral. A one-way Chi-squared analysis was conducted using the paw preference scores to determine whether there was any difference in the number of cats classed as left-pawed, right-pawed or ambilateral.

Binomial tests were subsequently performed to investigate whether or not there was a significant difference in the number of cats that were classified as exhibiting a paw preference (left or right) and those that were ambilateral. Further binomial tests on the paw-preferent cats explored whether there was a significant difference in the number of animals that were right- versus left-paw preferent.

A directional handedness index (HI) was calculated (see [35]) to quantify each cat’s lateral preference on the task on a continuum from strongly left-side preferent (+1) to strongly right-side preferent (−1). The HI was calculated by dividing the difference between the total number of left and right side uses by their sum (L−R)/(L+R). A Kruskall–Wallis test was performed on the cats’ HI scores to determine whether the direction of the cats’ paw preferences differed between the various breeds (Ragdoll, Maine Coon, Persian, Bengal, moggy). Mann–Whitney U tests explored for sex (male, female) differences in the cats’ HI scores.

The strength of the cats’ lateral preferences was calculated by taking the absolute value of each HI score (ABS-HI) for each measure. Kruskall–Wallis analysis was conducted to explore the effect of the cats’ breed (Ragdoll, Maine Coon, Persian, Bengal, moggy) on their ABS-HI scores. The effect of the cats’ sex (male, female) on the strength of their lateral bias was determined using Mann–Whitney U tests.

### 2.5. Ethical Note

This study was granted full ethical approval by the Ethics Committee, School of Psychology, Queen’s University Belfast (No. 45-2014).

## 3. Results

### 3.1. Distribution of Paw Preference

Based on the z-score analysis, 25 (44.6%) cats were classified as left-pawed, 20 (35.7%) as right-pawed and 11 (19.6%) as ambilateral. This distribution of paw preference was not significantly (χ2[df = 2, *n* = 56] = 5.39, *p* = 0.07) different from that expected by chance alone. Significantly more cats had a paw preference (left or right) than were ambilateral (binomial test, *p* < 0.001), however, the paw-preferent cats were no more likely to be left- than right-pawed (*p* = 0.55, binomial test).

**Figure 2:**
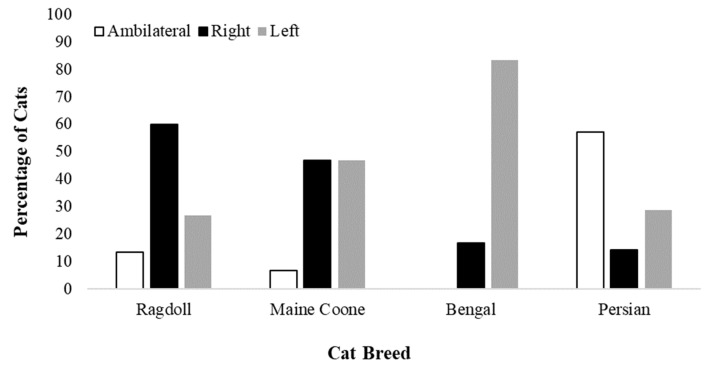
Distribution of paw preferences according to cat breed.

The distribution of the cats’ paw preferences differed significantly by breed (χ2[df = 6, *n* = 56] = 25.90, *p* < 0.001, Figure 2). One-way Chi-squared tests showed no significant difference in paw preference distribution for the Ragdolls (χ2[df = 2, *n* = 15] = 5.20, *p* = 0.07), Maine Coons (χ2[df = 2, *n* = 15] = 4.80, *p* = 0.09) or Persians (χ2[df = 2, *n* = 14] = 4.0, *p* = 0.13). Bengal cats, however, showed a highly significant deviation in their paw preference from that expected by chance alone (χ2[df = 2, *n* = 12] = 14.00, *p* < 0.001), with animals leaning more towards left- than right-pawedness or ambilaterality.

The propensity of the cats to be paw-preferent vs. ambilateral also differed significantly by breed (χ2[df = 3, *n* = 56] = 17.38, *p* = 0.001). Binomial tests showed that Bengals (*p* < 0.001), Ragdolls (*p* = 0.007) and Maine Coons (*p* = 0.001) were significantly more likely to be paw-preferent than ambilateral. Persian cats did not differ significantly in their propensity to be paw-preferent vs. ambilateral (*p* = 0.79).

### 3.2. Direction of Paw Preference

A Kruskal–Wallis test revealed no significant relationship between feline breed and the cats’ HI scores (χ2(3) = 6.96, *n* = 56, *p* = 0.07). However, results showed a significant effect of feline sex on the direction of the cats’ paw preferences (Mann–Whitney U = 223.50, n1 = 32 n2 = 24, *p* = 0.008). Males were significantly more likely to use their left paw on the task (mean HI = 0.42 ± 0.11), while females were more inclined to use their right paw (mean HI = −0.05 ± 0.10).

### 3.3. Strength of Paw Preference

Analysis revealed a significant effect of feline breed on the strength of the animals’ paw preference (χ2(3) = 15.60, *n* = 56, *p* = 0.001). Post-hoc tests showed that Persians had a significantly lower strength of paw preference score (mean ABS-HI = 0.30 ± 0.06) than Ragdolls (Mann–Whitney U = 32.00, n1 = 14, n2 = 15, *p* = 0.001; mean ABS-HI = 0.63 ± 0.06), Maine Coons (Mann–Whitney U = 34.50, n1 = 14, n2 = 15, *p* = 0.001; mean ABS-HI = 0.63 ± 0.06) and Bengals (Mann–Whitney U = 146.50, n1 = 14, n2 = 12, *p* = 0.001; mean ABS-HI = 0.68 ± 0.07).

There was no significant effect of feline sex on the strength of the animals’ paw preferences (Mann–Whitney U = 267.50, n1 = 32 n2 = 24, *p* = 0.06).

## 4. Discussion

The findings from this study point to breed differences in lateralised behaviour in the domestic cat and lend further support for the feline sex effects reported elsewhere.

Most of the animals in the present study showed a lateral bias on the food-reaching task designed to measure paw preferences. Paw-preferent animals, however, did not differ significantly in their tendency towards left- or right-pawedness. Previous studies have reported a roughly similar distribution of lateralisation in cats tested on a range of paw preference challenges [30,31,47,48], including the same task employed here [28,29].

The distribution of the cats’ paw preferences was found to be significantly related to feline breed. Maine Coons, Ragdolls and Bengals were significantly more likely to be paw-preferent than ambilateral, although only the Bengal cats showed a consistent preference for the use of one limb over the other. Nearly all the cats of this breed (83.3%) showed a left-sided paw preference, hinting at right hemisphere dominance. The right hemisphere has been linked to aggressive tendencies in several species. For example, Anolis lizards show a preference for using their left eye during aggressive interactions [49], while domestic dogs have been shown to turn their heads more towards threatening stimuli (silhouettes of snakes and threatening cats) that are presented on their left- as opposed to their right-hand side [50]. Dogs have also been shown to wag their tails more to the left-hand side when presented with visual stimuli (e.g., unfamiliar person) that might be expected to elicit withdrawal tendencies [51,52]. The Bengal cat has been ranked high for traits including aggression [41] and predatory behaviour [43]. The results from the present study lend support for emotional reactivity in the Bengal and are in keeping with the emotional valence theory of laterality [53] in suggesting that aggression is under the control of the right hemisphere.

The strength of the cats’ paw use was also related to breed. Persian cats were found to be more weakly lateralised than the other breeds recruited for this investigation, which, by contrast, were more likely to exhibit a left- or right-sided motor bias (strongly so in the case of the Bengal, see earlier). In humans, the preferred use of one hand has been linked to increased activity in the contralateral hemisphere [54]. Research has shown that the two cerebral hemispheres control very different functions, one being emotional reactivity; however, the exact influence of each hemisphere in emotional processing is still debated (for a review see [53]). The left hemisphere is largely believed to dominate approach and exploratory behaviour, whilst inhibiting fear [6]. The right hemisphere, by contrast, is thought to control the processing of fear and stimulates withdrawal in novel situations [55]. The dominant use of one hemisphere over the other predisposes an individual to behave in a certain way. Research has shown that weakly lateralised individuals (i.e., those who do not show a strong left or right hand preference for particular activities—and who thus do not rely on the dominant use of one hemisphere) are more likely to be fearful and susceptible to maladaptive behaviour than right-handed or strongly lateralised individuals [56]. For instance, research with dogs has shown that those displaying ambilateral paw preferences are more emotionally reactive to noise (sounds of thunderstorms) than paw-preferent dogs [57] (although, see [58]), while ambilateral chicks emit more distress calls in response to a predator than their lateralised counterparts [59]. Hart and Hart [41] ranked the Persian cat very highly on the trait of fearfulness to unfamiliar humans, suggesting that this breed has an emotionally reactive disposition. The fact that the Persian cats in this study were more likely to show an ambilateral paw preference would lend support for emotional reactivity in this breed. That said, a recent owner-assessed evaluation of breed-specific characteristics of pet cats did not unearth emotionally reactive dispositional traits for the Persian, instead highlighting lower scores (as assessed by the Fe-BARQ, a tool designed to assess feline temperament) for playfulness, predatory behaviour and prey interest [43]. Research on breed-related differences in the domestic cat would possibly benefit from assessments using multiple measures of individuality, which, taken together, might offer more rounded breed-related profiles than those arising from the exclusive use of one tool over another.

The direction of the cats’ paw preferences was found to be unrelated to feline breed but was very significantly associated with the animals’ sex, with male cats being more inclined to use their left paw, and females veering more heavily towards a right-sided bias. This sex split has been found previously in cats [28,29,30,31] and other species, e.g., dogs [38,60] and horses [37]. It is interesting that a sex effect was actually discovered in this study, since the sample was mostly (62.5%) comprised of castrated animals. Studies on dogs have failed to report an effect of canine sex on paw preferences in samples of neutered, or a mixture of de-sexed and entire, animals [36,55,61,62]; this has led the authors to argue that a hormonal factor may be at play in shaping motor bias. That said, McDowell and colleagues [29] recently found a very strong sex effect on a range of tasks exploring lateral bias in cats, using a population of entirely neutered subjects. The strong sex effect reported here, and elsewhere, using both castrated and de-sexed populations, points more and more strongly to underlying differences in the neural architecture of male and female cats. This is perhaps not surprising, given the sex differences in brain asymmetries reported across species [63].

## 5. Conclusions

Overall, the findings from this study point, for the first time, to an association between breed and lateral bias in the domestic cat. Cat breeds, unlike other domesticated species (e.g., dogs, horses), do not differ greatly in their morphology nor have they been bred by humans to serve different functions; they do, however, differ in their behavioural traits, e.g., personality [42]. Previous work has already demonstrated a correlation between lateral bias and temperament in the domestic cat [28], and the findings from the present study add to our understanding of this association. The results are largely in keeping with the emotional valence theory of laterality [51], with breeds prone to emotionally reactive dispositions (bearing in mind that studies of such dispositions are not in complete concordance) displaying different patterns of paw use with respect to those with less reactive temperaments. Assessing the paw preferences of different cat breeds alongside more traditionally employed assessments of breed-specific characteristics (e.g., owner-assessed personality questionnaires [43], expert opinions [41,42]), would help to build a bigger picture of breed-related dispositional traits. Such information would be of benefit to individuals considering the acquisition of a new cat, possibly helping to reduce disparities between owner expectations and pet behaviour; this may lead to more successful pet-owner relationships, reduced relinquishments and enhanced feline welfare. Further work, using larger numbers of subjects and a wider variety of breeds, is recommended.
